# Alteration of the Cardiac Sympathetic Innervation Is Modulated by Duration of Diabetes in Female Rats

**DOI:** 10.1155/2011/835932

**Published:** 2011-07-17

**Authors:** Jitka Švíglerová, Jiří Mudra, Zbyněk Tonar, Jana Slavíková, Jitka Kuncová

**Affiliations:** ^1^Department of Physiology, Faculty of Medicine in Plzeň, Charles University, Lidická 1, 301 00 Plzeň, Czech Republic; ^2^Department of Histology and Embryology, Faculty of Medicine in Plzeň, Charles University, Karlovarská 48, 301 00 Plzeň, Czech Republic

## Abstract

To evaluate the sympathetic innervation of the female diabetic heart, resting heart rate and sympathetic tone were assessed in vivo, and effect of tyramine on spontaneous beating rate, norepinephrine atrial concentrations, uptake, and release were determined in vitro in streptozotocin- (STZ-) treated rats and respective controls aged 3 months to 2 years. Resting bradycardia, decreased sympathetic tone, deceleration of spontaneous beating rate, and slightly declining carrier-mediated, but preserved exocytotic norepinephrine release from the atria were found in younger diabetic rats while the reactivity of the right atria to tyramine was not affected with age and disease duration. Diabetic two-year-old animals displayed symptoms of partial spontaneous recovery including normoglycemia, increased plasma insulin concentrations, fully recovered sympathetic tone, but putative change, in releasable norepinephrine tissue stores. Our data suggested that female diabetic heart exposed to long-lasting diabetic conditions seems to be more resistant to alteration in sympathetic innervation than the male one.

## 1. Introduction

Cardiovascular autonomic neuropathy (CAN) represents the most clinically important form of diabetic autonomic neuropathy. CAN results from damage to the sympathetic and parasympathetic innervation of the cardiovascular system, and it is typically manifested by reduced heart rate variability, exercise intolerance, orthostatic hypotension, and silent myocardial ischemia [1]. Autonomic dysfunction manifested by intracardiac sympathetic imbalance with predisposition to arrhythmias has been proposed as a cause of the increased incidence of sudden cardiac death in diabetic patients [2]. 

Although multiple data indicated an abnormal activity of the sympathetic nervous system in both diabetic human patients and animals with various experimental models of diabetes, their results are contradictory showing decrease, increase, or no change in plasma catecholamine levels, cardiac norepinephrine content, release, or uptake [3, 4]. The regulation of norepinephrine tissue stores is very complex involving control of expression and/or activity of the key synthesizing enzyme tyrosine hydroxylase, catecholamine exocytotic and nonexocytotic release, its uptake, and metabolism [5–7]. The inconsistent results of studies dealing with norepinephrine turnover in the tissues of streptozotocin- (STZ-) diabetic animals were attributed mostly to the duration and severity of diabetes [8]. 

Remarkably little attention has been paid to this issue in female rats, although multiple lines of evidence suggest considerably higher diabetes-related relative risk for serious cardiovascular complications in diabetic women than in men [9, 10]. Recently, we have reported an interesting feature of STZ-induced model of diabetes in female rats: within one year of the disease duration, a substantial number of STZ-injected animals displayed symptoms of partial spontaneous recovery with decreasing blood glucose concentrations and increasing insulin levels in the plasma and pancreas [11]. However, despite this spontaneous improvement of the diabetic state, norepinephrine levels in the heart compartments remained lower than in the age-matched controls 12 months after the onset of the disease. 

The aim of the present study was to investigate how diabetes affects the content, release, and uptake of norepinephrine in cardiac sympathetic nerves 1 to 22 months after STZ administration (i.e., at the ages from 3 months to 2 years) in female rats with severe uncompensated diabetes, in animals that displayed symptoms of partial spontaneous recovery, and in the age-matched controls. 

## 2. Materials and Methods

### 2.1. Animals

Wistar female rats purchased from VELAZ (Prague, Czech Republic) at the age of 50 days were used. The animals were housed five per cage and fed standard laboratory chow with free access to drinking water. All animals were left intact to adapt for 2 weeks before the initiation of the study. All experiments were conducted in accordance with the European Directive for the Protection of Vertebrate Animals Used for Experimental and Other Scientific Purposes (86/609/EU) and the relevant guidelines of the Czech Ministry of Agriculture for scientific experimentation on animals and were approved by the University Committee for Experiments on Laboratory Animals. After adaptation, rats (*n* = 270) were randomly divided into two groups: 160 animals were rendered diabetic by a single intravenous injection of STZ (65 mg/kg of the body weight) dissolved in citrate buffer (pH 4.5), and 110 animals were injected with vehicle serving as controls. Diabetes was verified by severe hyperglycemia 48 hours after STZ administration. Blood glucose levels were measured by glucose oxidase method and checked bimonthly till the age of one year and then 22 months after the onset of the disease. About 30%  (*n* = 46) of diabetic rats died in the course of the study. The rats were used for further experiments at 1, 2, 4, and 22 months after the induction of diabetes or vehicle administration and designated STZ1, STZ2, STZ4, and STZ22 and CONT1, CONT2, CONT4, and CONT22, respectively. 

### 2.2. Measurement of the Heart Rate

To estimate the tonic influence of the cardiac sympathetic innervation on heart rate, the animals were placed in a small chamber with electrodes in the floor that were connected to an electrocardiograph (EKG Seiva Praktik, Prague, Czech Republic). Animals were left to adapt for 20 min, and then the resting heart rate was measured five times in one-minute intervals. The effect of nonselective *β*-adrenergic receptor antagonist metipranolol (2 mg/kg b.w., i.p.) on heart rate was tested after previous administration of atropine (4 mg/kg b.w., i.p.). First, atropine was administered; rats were left to adapt for 15 min; their heart rates were measured five times, and then metipranolol was injected, and heart rate was measured again 5 times after 15 min adaptation period. The values presented in the results represent means of these measurements. 

### 2.3. Glucose Tolerance Tests

Glucose tolerance tests were performed in 24-month-old diabetic and age-matched control rats (*n* = 5). The rats were fasted from evening before and anaesthetized with urethane 1.5 g/kg b.w. Glucose 2 g/kg b.w. was administered intraperitoneally, and blood samples were collected from the orbital sinus before and at 15, 30, 60, 90, 120, and 180 min after the glucose challenge (Figure 2). 

### 2.4. Determination of Norepinephrine and Insulin Tissue Concentrations

Diabetic rats of all categories and their respective controls were anaesthetized with ether, killed by cervical dislocation, and exsanguinated. Blood was collected on EDTA and centrifuged (1000 ×g, 10 min, 4°C). The aspirated plasma was stored at −20°C for subsequent determination of insulin concentration. The dissected right and left heart atria and pancreas were rinsed with ice-cold saline, immediately frozen on dry ice, and weighed. Norepinephrine was extracted from the atria by homogenization in 10 volumes of 0.1 mol/L HCl and subsequent centrifugation (5000 ×g, 20 min, 4°C). Supernatants were diluted 1 : 10 with distilled water and stored at −70°C until radioimmunoassay (RIA). Norepinephrine concentrations in the tissue extracts were measured by commercial diagnostic kits (IBL, Hamburg, Germany).

Pancreatic tissues were frozen, weighed, and homogenized at 4°C in 10 volumes of acid-alcohol solution (75%  (vol/vol) ethanol, 23.5% bidistilled water, and 1.5%  (vol/vol) 10 mol/L HCl). Homogenates were centrifuged (5000 ×g, 20 min, 4°C); the supernatants stored at −20°C until RIA for insulin measurements (kits LINCO Research, St. Charles, Mo, USA). 

### 2.5. Immunohistochemistry of the Pancreas

For the immunohistochemical study, whole pancreata were fixed by immersion in 4% buffered formaldehyde, embedded in paraffin, and cut to 5 *μ*m thick tissue sections. The endogenous peroxidase activity was blocked by a solution composed of hydrogen peroxide (1 volume) and methanol (50 volumes). For detection of insulin, sections were incubated with polyclonal guinea pig anti-insulin (1 : 150; Dako, Carpinteria, Calif, USA) for 12 hours at 4°C. The secondary antibody (incubation for 45 min at 37°C) and avidin-biotin peroxidase complex (45 min, 37°C) were applied, using the Novostain Super ABC Universal Kit (Novocastra Laboratories Ltd., Newcastle upon Tyne, UK). Following the immunohistochemistry, the background tissue was stained with Gill's haematoxylin (30 s; Bio-Optica, Milano, Italy).

For the quantification of insulin-positive cells, the volume fraction of insulin-positive cells within the pancreas *V*_*v*_(insulin, pancreas) was estimated in two CONT22, two STZ1 and five STZ22 rats according to the following equation:

(1) $$\begin{array}{c} V_{v}\left(\text{insulin},\text{pancreas}\right)=\frac{\text{est}V\left(\text{insulin}\right)}{\text{est}V\left(\text{pancreas}\right)}, \end{array}$$

where est*V*(insulin) is the estimated volume of cytoplasm of cells stained with the insulin antibody, and est*V*(pancreas) is the total volume of the pancreas. Each pancreas was cut exhaustively to a series of 5-*μ*m-thick histological sections and every eighth section, was selected for quantification, using systematic uniform random sampling. After taking calibrated micrographs, we assessed the area of the profiles of insulin-positive cells and area of whole pancreatic tissue using the stereological point-counting method, see the following equation:

(2) $$\begin{array}{c} \text{est}A=a\cdot P, \end{array}$$

where est*A* is the estimated area of profiles of insulin-positive cells (or the whole pancreas, resp.), grid parameter *a *is the area corresponding to one test point according to the calibration, and *P* is the number of test points hitting the insulin-positive cells (or the whole pancreas, resp.). The total number of points counted for both insulin-positive cells as well as the whole pancreas was at least 200 in each series of sections. We used the Cavalieri principle for estimating the volume est*V*(insulin) and the est*V*(pancreas), see the following equation:

(3) $$\begin{array}{c} \text{est}V=T\cdot \left(A_{1}+A_{2}+\cdots +A_{m}\right) \end{array},$$

where est*V* is the Cavalieri volume estimator, *T* = 0.040 mm is the distance between the two following selected sections, *A*_*i*_ is the area of the insulin-positive cells (or the pancreas, resp.) in the *i*th section, and *m* is the total number of sections selected from the series. Stereological analysis was done using the PointGrid module of the Ellipse software (ViDiTo, Kosice, Slovakia). 

### 2.6. Evaluation of Tyramine-Induced Positive Chronotropic Effect

The chronotropic experiments were performed 1, 2, 4, and 22 months after STZ or citrate buffer application. Animals were anaesthetized with intraperitoneal injection of urethane (1.5 g/kg body weight) 10 min after having received heparin (500 U, i.p.), and their hearts were quickly excised. The right atria were snipped and placed into an experimental chamber with Tyrode solution. The solution was maintained at 32°C and continuously aerated with clear oxygen. The action potentials of the preparation were recorded with an extracellular electrode, which was attached to the preparation and with the second electrode freely placed in the solution without any contact with the preparation. Potentials were recorded by means of the laboratory system Biopac (Biopac Systems, Inc.; Goleta, CA, USA). After stabilization period (at least 30 min), the chronotropic effect of tyramine was tested. The cumulative doses of tyramine (concentrations from 10^−7^ to 10^−4^ mol/L) were administrated at two-minute intervals. The potentials were recorded at the end of two-minute interval, and higher concentration of tyramine was added immediately afterwards. The heart rate was calculated as the average of five randomly chosen values from each recording. 

### 2.7. Norepinephrine Release from the Atria

**Figure 1:**
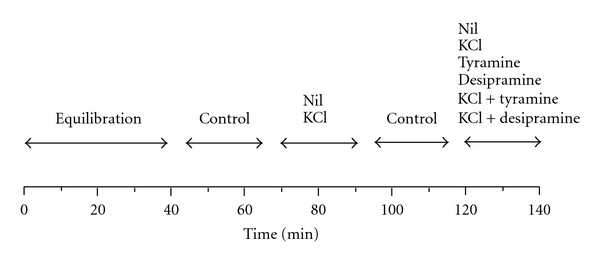
Scheme of the superfusion experiments. One intervention per experiment was assessed.

Control and diabetic rats were anaesthetized with ether and decapitated. The hearts were rapidly excised and placed into ice-cold Tyrode solution containing 1 *μ*mol/L monoaminooxidase inhibitor pargyline. Both atria were dissected, sliced into pieces (thickness 300 *μ*m) by McIllwain tissue chopper, and pooled in a beaker containing oxygenated Tyrode solution with pargyline. Tissue slices (approx. 50–100 mg of atrial tissue) were transferred into 12 parallelly perfused 0.5 mL perfusion chambers. Tissues were superfused at a rate of ~0.1 mL/min with oxygenated Tyrode solution with pargyline. All perfusion experiments were performed at 37°C. After 40 min equilibration period, four 20 min fractions were collected at 0°C according to the scheme in Figure 1.

In all perfusion experiments, the first 20 min fraction served to compare catecholamine releases among various perfusion experiments. The effects of tyramine (10^−6^ mol/L) and desipramine (10^−7^ mol/L) on basal and K^+^-stimulated releases of norepinephrine from the sliced atria were tested. Basal release was determined as the catecholamine outflow from the atria perfused with oxygenated Tyrode solution with pargyline in the second 20 min fraction. K^+^-stimulated release was taken as norepinephrine output from tissues stimulated by depolarization with 50 mmol/L KCl with a concomitant reduction of NaCl in the superfusion fluid in the second fraction. The third fraction was obtained after perfusion with Tyrode solution and served to check for consistency of experiments. Then, the perfusion solution was changed for Tyrode solution with desipramine, tyramine when basal release was tested, or K^+^-rich solution with these substances when their effects on stimulated release were assessed. 

In the additional experiments, the tissue slices were superfused with basal or K^+^-rich solutions with above-mentioned substances added (i.e., tyramine and desipramine) for 2 hours. Before and after the perfusion, tissues were transferred into pre-weighed test-tubes, centrifuged at 5000 ×g, 4°C for 15 min, supernatants were discarded, the samples were weighed and stored at −70°C for subsequent determination of catecholamine concentration in tissue slices before and after perfusion. Norepinephrine concentrations in the superfusates were determined using radioimmunoassay ultrasensitive diagnostic kits (LDN, FRG). 

### 2.8. Solutions and Chemicals

The composition of the Tyrode solution was following (in mmol/L): NaCl 137, KCl 4.5, MgCl_2_ 1, CaCl_2_ 2, glucose 10, Hepes 5; pH was adjusted to 7.4 with 1 mol/L NaOH. Metipranolol was from Hoechst-Biotika, Martin, Slovac Republic. Atropine, streptozotocin, tyramine hydrochloride, desipramine hydrochloride were purchased from Sigma Aldrich (Prague, Czech Republic). Other chemicals were from Lachema (Brno, Czech Republic). All chemicals were of analytical grade. 

### 2.9. Data Analysis

Results are presented as means ± SEM. Statistical differences were tested by unpaired two-tailed Student's *t*-test or by analysis of variance (ANOVA) followed by post hoc Fisher's Least Significant Difference test, using software package STATISTICA Cz, version 7 (StatSoft, Inc., Tulsa, OK, USA). Normality of populations and homogeneity of variances were tested before each ANOVA. The results were considered significantly different when *P* < 0.05. 

## 3. Results

### 3.1. General

**Figure 2:**
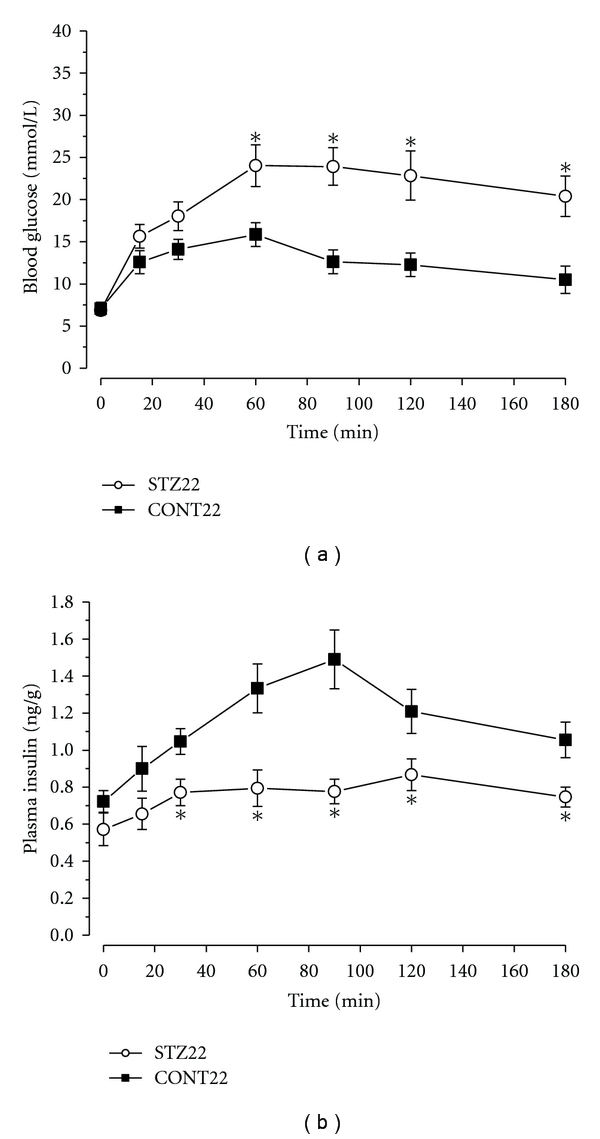
Glucose tolerance tests. Response to intraperitoneal administration of glucose (2 g/kg) in old diabetic (STZ22) rats and their age-matched controls (CONT22). Values are means ± SEM (*n* = 5 per group). (a) Blood glucose concentrations in given time intervals after glucose administration. (b) Plasma insulin concentrations after the glucose challenge. *significantly different from value in control group (*P* < 0.05).

Prior to STZ administration, there were no significant differences in the body weights, blood glucose levels, and plasma insulin concentrations between the animals subjected to STZ or vehicle injection. When compared to controls, body weights, plasma and pancreatic insulin levels were significantly lower (*P* < 0.01), and blood glucose concentrations significantly higher in diabetic rats 1, 2, and 4 months after STZ administration (Table 1). Interestingly, animals 22 months after STZ administration displayed symptoms of partial spontaneous recovery having blood glucose and plasma insulin levels not differing from age-matched controls. However, body weights remained significantly lower than in the age-matched controls (*P* < 0.05) and their glucose tolerance was markedly impaired as shown in Figure 2. 

**Figure 3:**
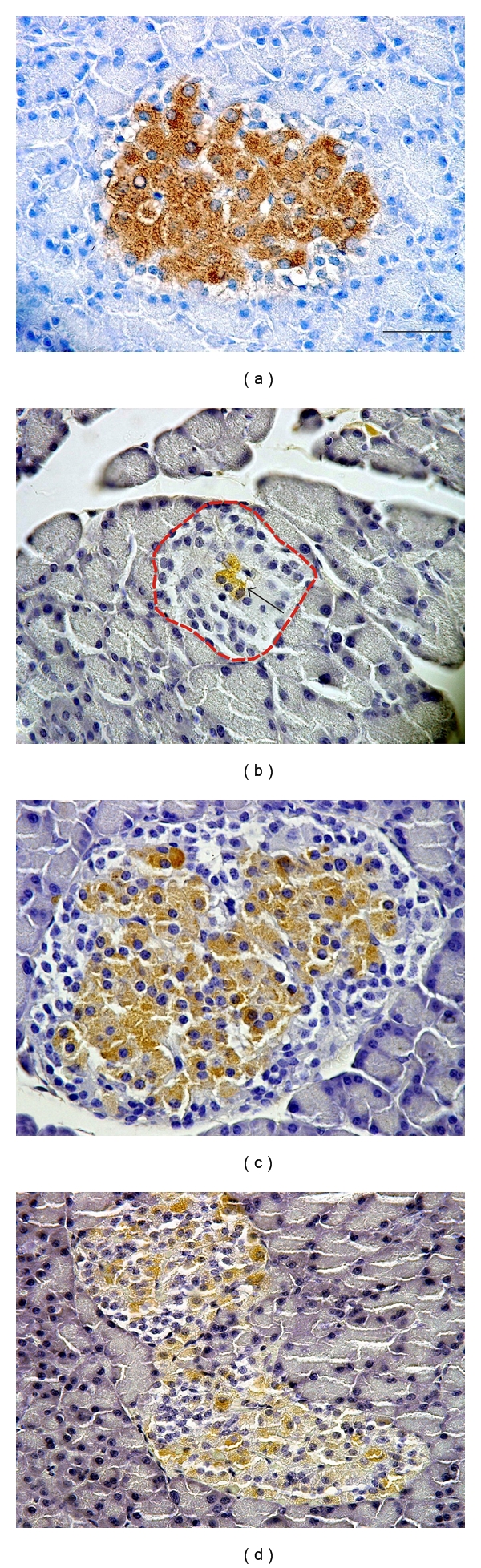
Expression of pancreatic insulin detected by immunohistochemistry in representative micrographs of a young healthy rat (a), a diabetic rat 1 month after STZ administration (b), an old healthy rat (c), and an old diabetic rat 22 months after STZ administration (d). In (a), the islets were rich in insulin-positive cells; also the intensity of reaction was very strong. In (b), the islets (red-dashed outline) were small and contained only traces of insulin positivity (green arrow). The overall shrinkage of islet mass and depopulation of beta cells in group were accompanied by islet reorganization, impairing the natural structure of endocrine trabecular epithelium. In (c), the islets were of normal size, while the insulin-positive cells occupied most of the islets. In (d), the islets showed signs of tissue remodelling when compared with control rats, but they contained population of insulin-positive cells similar to the pancreata of healthy animals of comparable age. Scale bar indicates 50 *μ*m.

In all samples processed by immunohistochemistry, the pancreata were well preserved, without autolysis and islets showed no signs of either florid inflammation or lymphocytes/macrophages infiltration. Typical histological findings are summarized in Figure 3. Quantitative analysis of the selected pancreatic samples indicated that volume fraction of insulin-positive cells within the pancreas *V*_*v*_ (insulin, pancreas) of CONT22 rats was 0.15 ± 0.01%, which was significantly higher than in STZ1 rats (0.02 ± 0.01%; *P* < 0.05). In STZ22 samples, volume fraction of insulin-positive cells reached the value 0.08 ± 0.03%, that is, higher than in STZ1 rats, but still lower than in CONT22 pancreata. 

### 3.2. Effect of Metipranolol after Atropine Pretreatment on the Heart Rate

As shown in Table 2, STZ-diabetic females displayed significant resting bradycardia from the first month after the onset of the disease with a trend to further reduce the heart rate with the disease duration. In addition, resting bradycardia was accompanied with an almost complete loss of the sympathetic nervous tone to the heart. Partial spontaneous recovery of diabetes at the age of 2 years led to full restoration of the sympathetic tone; however, resting bradycardia was still considerable and the spontaneous beating rate of the isolated heart atria was the slowest in these aged STZ-diabetic animals compared to all younger diabetic and age-matched control rats (Figure 5). Thus, in spite of better diabetes compensation, the function of sinoatrial node further worsened in the course of experiment.

### 3.3. Norepinephrine Concentrations in the Heart Atria of Control and Diabetic Rats

**Figure 4:**
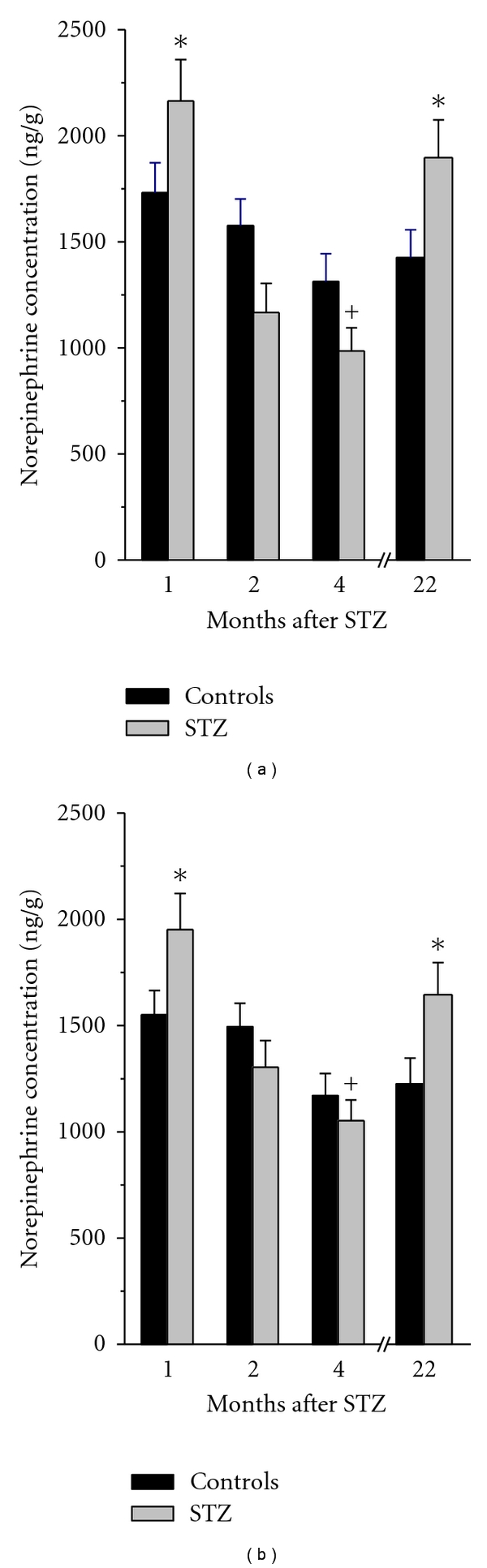
Norepinephrine concentrations in the right (a) and left (b) atria of STZ-diabetic rats 1, 2, 4, and 22 months after STZ administration and their respective controls. Data are means ± SEM; *P < 0.05 compared to the respective control; **^+^** *P* < 0.05 compared to STZ1 value.

In the control rats at the age of 3 months, norepinephrine concentrations were 1731 ± 142 ng/g and 1550 ± 115 ng/g in the right and left atria; respectively, and they did not change significantly with age. The diabetic state led to a slight, but significant, increase in atrial norepinephrine levels 1 month after STZ administration. In STZ2 and STZ4 rats, norepinephrine concentrations in both atria did not significantly differ from the respective controls although they displayed declining trend compared to STZ1 animals. In contrast, STZ22 rats had atrial tissue concentrations of norepinephrine by ~30–40% higher than the age-matched controls (Figure 4). 

### 3.4. Evaluation of Tyramine-Induced Positive Chronotropic Effect

**Figure 5:**
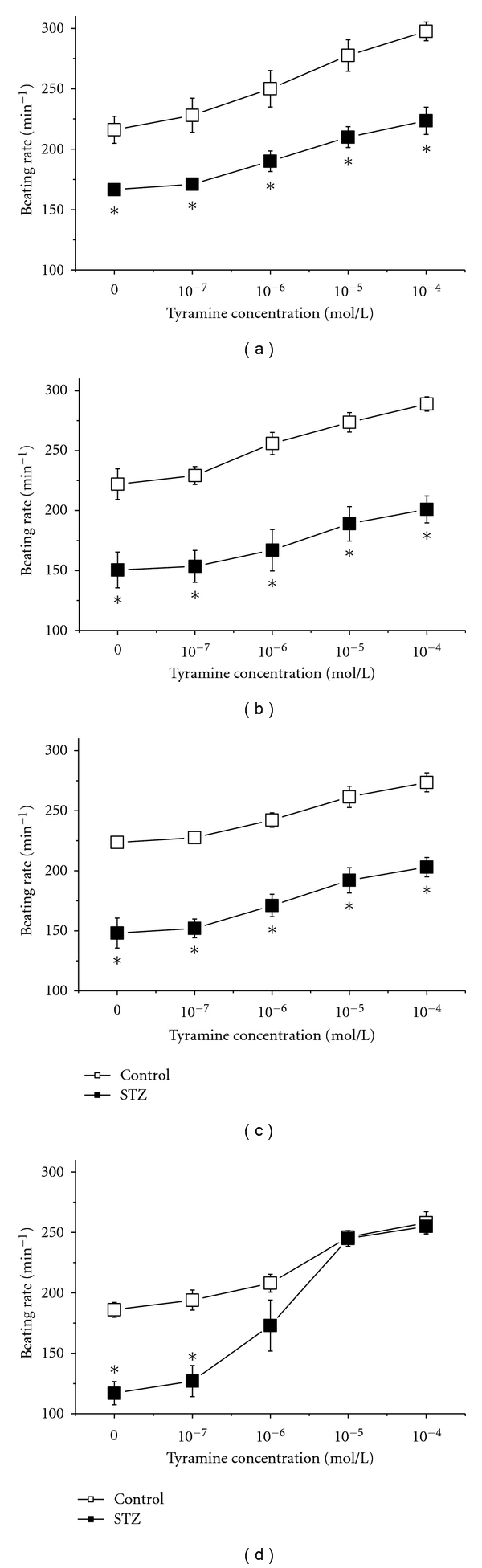
The effect of tyramine on the spontaneous beating right atria in control and diabetic (STZ-) rats 1 (a), 2 (b), 4 (c) and 22 (d) months after STZ or vehicle application. Open squares—control rats (*n* = 5 in (a, (b),and (c)), *n* = 4 in (d), filled squares—diabetic rats (*n* = 5 in panels a, b, and c, *n* = 4 in panel d). *significantly different from control rats (*P* < 0.05).

The spontaneous beating rate of the isolated right atrium in tyramine-free solution was significantly lower in diabetic rats compared to control ones in all age categories. The beating rate in control groups decreased with age to values significantly lower in CONT22 samples as against all younger age categories reaching 216 ± 11 beats/min in CONT1 and 186 ± 6 beats/min in CONT22 atria. This time-dependent decrease in the spontaneous beating rate was yet more pronounced in diabetic rats, where it was significantly lower in partially compensated diabetic rats compared to younger STZ groups reaching 166 ± 4 beats/min in STZ1 samples and 117 ± 10 beats/min in STZ22 ones (Figure 5).

In both control and diabetic groups, tyramine (stimulator of norepinephrine release via inversely working norepinephrine transporter) exerted the dose-dependent positive chronotropic effect on the spontaneous beating rate of the right atrium. The dose-related increment in the pacemaker discharge rate was comparable in all control groups (Figure 5). In uncompensated diabetic groups, the concentration dependent chronotropic effect of tyramine did not show any important difference compared to age-matched controls; beating rate in STZ1, STZ2, and STZ4 at tyramine concentration of 10^−4^ mol/L was by ~35% higher compared to the rate in tyramine-free solution (Figures 5(a), 5(b), and 5(c)). In contrast, the isolated atria of partially compensated rats displayed a steep increase in sensitivity to chronotropic effect of tyramine; discharge rate in STZ22 at tyramine concentrations from 10^−6^ to 10^−4^ mol/L did not differ from age-matched controls (Figure 5(d)). 

### 3.5. Release of Norepinephrine from the Heart Atria

**Figure 6:**
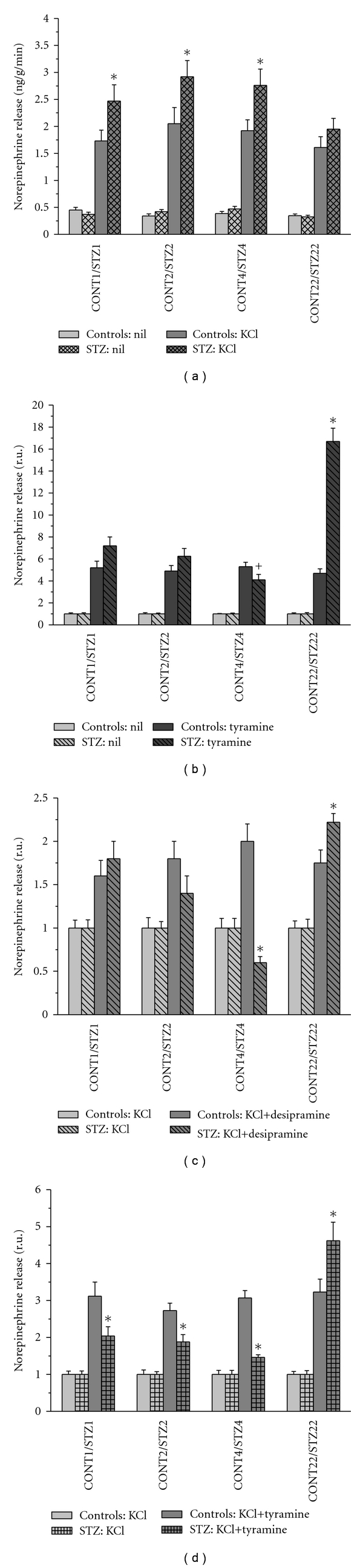
Norepinephrine release from the sliced heart atria of control and diabetic (STZ) rats 1, 2, 4, and 22 months after STZ or vehicle application (CONT1, CONT2, CONT4, and CONT22 and STZ1, STZ2, STZ4, and STZ22, resp.). Data are means ± SEM; *P < 0.05 compared to the respective control; **^+^** *P* < 0.05 compared to STZ1 value.

Basal release of norepinephrine from the control atria was 450 ± 35 pg/g/min and it was not significantly affected by the age of animals, being 348 ± 42 pg/g/min, in CONT22 rats. Basal norepinephrine release from the atrial preparations of diabetic rats did not differ from the respective control values. Norepinephrine release evoked by depolarization with high KCl concentration was increased in STZ1, STZ2, and STZ4 atria when compared to the respective controls. In contrast, STZ22 samples displayed KCl-evoked norepinephrine release not differing from the control preparations (Figure 6(a)). Tyramine significantly enhanced basal norepinephrine release from both control and diabetic atria; however, in STZ4 samples it was significantly lower than that in STZ1 ones (Figure 6(b)). Desipramine, blocker of neuronal norepinephrine transporter, had no significant effect on basal norepinephrine overflow from both control and STZ-treated rat heart atria (data not shown). In contrast, it enhanced KCl-evoked release in all control preparations and STZ1, STZ2, and STZ22 atria, but inhibited the same parameter in STZ4 atrial preparations (Figure 6(c)). Tyramine in KCl-rich solution further increased norepinephrine output in all samples; however, its effect seemed to be relatively smaller in the diabetic rats 1, 2, and 4 months after the onset of diabetes and greater in STZ22 atria (Figure 6(d)). Nevertheless, when expressed in absolute values, there were no significant differences between tyramin-induced effects in K^+^-rich solution between control and diabetic preparations with exception of the STZ22 samples. 

### 3.6. Concentration of Norepinephrine in the Atrial Slices before and after Perfusion

**Figure 7:**
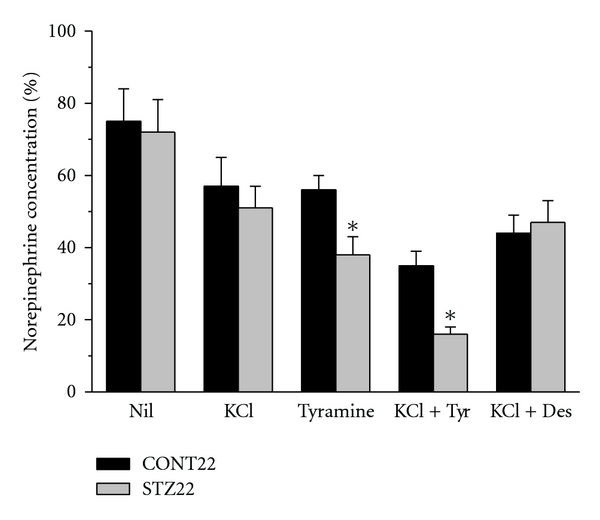
Concentrations of norepinephrine in tissue slices of the heart atria of control and STZ-diabetic rats at the age of 2 years (CONT22 and STZ22, resp.) after the perfusion experiments without (nil) or with releasing substances added. The data are expressed in % of the respective concentration before perfusion. Tyr: tyramine, Des: desipramine; *significantly different from control samples (*P* < 0.05).

To evaluate the releasable pool of norepinephrine in the heart atria, norepinephrine concentrations were measured in the sliced samples before and after the perfusion experiment. In the control samples and in all young diabetic groups (STZ1, STZ2, and STZ4), the effects of individual interventions on the relative norepinephrine levels in the tissue after perfusion did not significantly differ. Both tyramine and KCl depleted the norepinephrine contents to similar extent (~50%) and their combination to even ~30%. In contrast, tyramine alone depleted norepinephrine from STZ22 tissue slices by 62% and high K^+^ concentration with tyramine even by 84% (Figure 7). 

## 4. Discussion

The present study addressed the impact of short- and long-term diabetes on norepinephrine release and uptake in the heart atria of female rats aged 3 months to 2 years in relation to functional parameters, that is, the tonic influence of the sympathetic nervous system on the heart rate and chronotropic response of the isolated atria to tyramine-stimulated norepinephrine release. 

As expected, diabetic state induced by STZ was associated with severe hyperglycemia and hypoinsulinemia and low insulin positivity in the pancreas of rats 1, 2, or 4 months after STZ administration. However, at the age of 2 years, surviving diabetic animals displayed symptoms of partial spontaneous recovery with normal blood glucose and plasma insulin levels, but impaired glucose tolerance. Volume fraction of insulin-positive cells within the pancreas in partially compensated rats was higher than that in uncompensated diabetic animals, but still lower than that in the respective controls. We have already reported this interesting feature of STZ-induced diabetes in female rats at the age of 14 months when about 30% of animals displayed regeneration of the insulin-secreting tissue [11]. This study shows that with advancing age, all surviving diabetic animals seemed to recover incompletely from STZ-induced damage to pancreatic B cells; nevertheless, their response to glucose load did not result in any clear improvement in insulin secretion. The spontaneous partial recovery from STZ-induced diabetic state has been proved also in newborn rats [12] and in adult rats treated by low doses of STZ [13]. The sensitivity to STZ may be also influenced by gender—the lower susceptibility to diabetogenic effect of STZ has been found in female [14] and castrated male mice [15] compared to uncastrated males. 

Our study also indicated that the resting heart rate and sympathetic tone were not markedly affected in female control rats till the age of two years. In contrast, diabetic animals displayed resting bradycardia that could be at least partly attributed to almost complete disappearance of the sympathetic tone from the first month of the disease. Similar findings were reported in diabetic male rats 1 to 14 weeks after STZ administration [16]. Interestingly, in spite of full recovery of the sympathetic tone in old diabetic rats (STZ22), the resting heart rate remained significantly lower than that in the age-matched controls suggesting direct diabetes-induced damage to the sinoatrial node. Analysis of the spontaneous beating rate of the right atrium isolated from aging control and diabetic rats showed that the function of sinoatrial pacemaker declined with age, and this trend was further accentuated by diabetes. The age-dependent changes in the node could result from electrophysiological disturbances, degenerative changes of the myocardium or of restriction of pacemaker cells [17–20]. Regional defect in the expression and/or electrophysiology of ion channels and the structural remodeling of gap junction connexin proteins found in the STZ-treated rats may underlie dysfunction of the sinoatrial node and bradycardia in the experimental insulin-dependent diabetes mellitus [21, 22]. In addition, the alteration of heart rate and pacemaker dysfunction are known to be risk factors of arrhythmias observed in diabetic patients [23]. Gender differences in the propensity for various types of cardiac arrhythmias in nondiabetic population [24, 25] and experimental animals [26] are well recognized. Putative gender-specific impact on susceptibility to arrhythmias in long-standing STZ diabetes remains to be precisely determined. 

Assessment of parameters of the cardiac sympathetic innervation at the level of effector organ showed an increase in norepinephrine concentrations in the heart atria 1 month after the onset of diabetes followed by a decrease later, which is in good agreement with previous studies on male and female rats [11, 27–29]. The initial increase in the cardiac norepinephrine levels could be attributed to different mechanisms that have been reported in several studies on rats 4–12 weeks after administration of a diabetogenic STZ dose: increased synthesis of norepinephrine, due to the enhanced activity of tyrosine hydroxylase [8], increased uptake of norepinephrine by adrenergic nerve terminals [30] or its diminished calcium-dependent exocytotic release [31]. However, all these data were collected on relatively young male rats. Reports dealing with the impact of age on gender-specific features of the cardiovascular adrenergic innervation suggest multiple differences in exocytotic release, uptake, metabolism, and norepinephrine sensitivity between males and females: whereas, in males, cardiac norepinephrine concentration, release, uptake, and turnover rate were repeatedly reported to be decreased with advancing age, females seemed to be more resistant to these changes [32, 33]. Our study also documents that till the age of 2 years, norepinephrine concentrations in the atria, neurotransmitter release, and uptake remained comparable between young- and old- age female categories. 

The results of the present study also documented that exocytotic release tested as K^+^-evoked norepinephrine outflow from the sliced atria was enhanced in all uncompensated STZ-diabetic female rats (i.e., STZ1, STZ2, and STZ4) rats in contrast to previously described attenuation of norepinephrine exocytosis in the male atria 8 to 12 weeks after the onset of diabetes [31]. It should be noted that calcium dependency of K^+^-evoked release could be modified by diabetes. In one of our previous studies, an increased contribution of calcium-independent, that is, carrier-mediated nonexocytotic mechanism to K^+^-evoked norepinephrine release was documented [34]. Therefore, we studied the effects of norepinephrine transporter substrate tyramine and its blocker desipramine on both basal and stimulated releases. The results of these experiments suggest that in the first two months of severe diabetes, norepinephrine transporter function was not significantly affected. However, 4 months after the onset of diabetes, both tyramine and desipramine effects were significantly smaller than that in younger diabetics, even if expressed in absolute values. Tyramine enters the nerve ending via norepinephrine transporter, replaces norepinephrine in the cytoplasm, and via monoamine vesicular transporter also in the synaptic vesicles. Increased cytoplasmic concentration of norepinephrine then causes reversal of norepinephrine transport across the cell membrane [35]. Lower effect of tyramine could be related to the impaired function of norepinephrine transporter or compromised tissue stores of releasable norepinephrine. Indeed, in the diabetic male rats, 4 weeks of STZ-diabetes were sufficient to induce substantial reduction in norepinephrine transporter expression in the heart [36]. Diminished effect of desipramine could be explained by decreasing activity or density of the neuronal carrier [37]. However, this interpretation might be misleading in case of substantial changes in conditions influencing transporter affinity and direction of action [38] that could be inversed by an enhanced intracellular concentration of norepinephrine or sodium ions [31, 39, 40]. Both decreased activity of Na^+^-, K^+^-ATPase leading to decrease in the transmembrane sodium concentration gradient and defective vesicular transport of norepinephrine resulting in the increased cytosolic norepinephrine concentrations were suggested in STZ-diabetic hearts [31, 41]. Whatever were the mechanisms underlying diminishing effects of tyramine and desipramine on norepinephrine release from the diabetic atria, tyramine-induced increases in the atrial beating rate were comparable in all uncompensated STZ groups (i.e., STZ1, STZ2, and STZ4), thus implying well-balanced sensitivity of the target tissue to the neurotransmitter released.

The most interesting data were obtained from rats 22 months after STZ administration. They indicated that sympathetic tonic influence on the heart rate was fully restored with partial compensation of diabetes and that depolarization-induced release of norepinephrine was well preserved in spite of the long-term disease duration. Enhanced tyramine-induced release along with the highest effect of tyramine on the beating rate of the isolated right atria and striking alterations to norepinephrine atrial concentrations before and after perfusion indicated change in the releasable pool of norepinephrine in the nerve endings. 

## 5. Conclusion

Taken together, our data show that female diabetic heart seems to be more resistant to diabetes-induced alteration in adrenergic neurotransmission than the male one—exocytotic K^+^-induced release from the heart atria was well preserved till 4 months of diabetes duration. Slight declining trend could be observed in carrier-mediated release of norepinephrine; however, reactivity of the isolated right atria to tyramine was not altered with age and disease duration. In spite of this relatively well-preserved parameters of adrenergic neurotransmission at the level of effector organ, the tonic sympathetic influence on the heart rate was nearly negligible even at the first month of diabetes, and the sinoatrial beating rate also displayed symptoms of gradually deteriorating function. Partial compensation of diabetes led to fully recovered sympathetic tone and putative change in releasable norepinephrine tissue stores. 

## Figures and Tables

**Table 1:** Body weights, blood glucose concentrations, and plasma insulin levels in diabetic and control female rats.

Months after STZ	Blood glucose (mmol/L)		Body weight (g)		Plasma insulin (ng/mL)	
	Control (*n* = 6)	STZ (*n* = 6)	Control (*n* = 6)	STZ (*n* = 6)	Control (*n* = 6)	STZ (*n* = 5)
1	8.7 ± 0.4	23.7 ± 1.4*	270 ± 12	195 ± 5*	0.81 ± 0.09	0.11 ± 0.02*
2	8.2 ± 0.5	26.2 ± 2.4*	292 ± 8	198 ± 6*	1.09 ± 0.15	0.17 ± 0.02*
4	8.9 ± 0.2	26.6 ± 1.5*	315 ± 9	188 ± 4*	1.11 ± 0.12	0.14 ± 0.02*
22	6.2 ± 0.5	8.5 ± 0.8^#^	350 ± 12	235 ± 10^∗#^	0.86 ± 0.09	1.08 ± 0.16^#^

Values are means ± SEM.; STZ: streptozotocin; *significantly different from value in control group (*P* < 0.05); ^#^significantly different from STZ1 value (*P* < 0.05).

**Table 2:** Resting heart rate and the effect of metipranolol after atropine administration on the heart rate in diabetic and control female rats.

Months after STZ	Resting heart rate (min^−1^)		Effect of MP after ATR (min^−1^)	
	Control (*n* = 6)	STZ (*n* = 6)	Control (*n* = 6)	STZ (*n* = 5)
1	345 ± 8	280 ± 6*	−122 ± 7	−10 ± 8*
2	352 ± 9	252 ± 7*	−130 ± 8	−8 ± 5*
4	355 ± 7	238 ± 7*	−92 ± 5	−1 ± 5*
22	345 ± 10	309 ± 11*	−116 ± 9	−105 ± 11^#^

Values are means ± SEM; STZ: streptozotocin; MP: metipranolol; ATR: atropine; *significantly different from value in control group (*P* < 0.05); ^#^significantly different from STZ1 value *P* < 0.05.

## References

[1] Vinik AI, Freeman R, Erbas T (2003). Diabetic autonomic neuropathy. *Seminars in Neurology*.

[2] Bergner DW, Goldberger JJ (2010). Diabetes mellitus and sudden cardiac death: what are the data?. *Cardiology Journal*.

[3] Hilsted J (1995). Catecholamines and diabetic autonomic neuropathy. *Diabetic Medicine*.

[4] Heyman E, Delamarche P, Berthon P (2007). Alteration insympathoadrenergic activity at rest andduring intense exercise despite normal aerobic fitness inlate pubertal adolescent girls with type 1diabetes. *Diabetes and Metabolism*.

[5] Esler M, Jennings G, Lambert G, Meredith I, Horne M, Eisenhofer G (1990). Overflow of catecholamine neurotransmitters to the circulation: source, fate, and functions. *Physiological Reviews*.

[6] Eisenhofer G, Esler MD, Cox HS (1990). Differences in the neuronal removal of circulating epinephrine and norepinephrine. *Journal of Clinical Endocrinology and Metabolism*.

[7] Goldstein DS, Cannon RO, Quyyumi A (1991). Regional extraction of circulating norepinephrine, DOPA, and dihydroxyphenylglycol in humans. *Journal of the Autonomic Nervous System*.

[8] Ganguly PK, Dhalla KS, Innes IR (1986). Altered norepinephrine turnover and metabolism in diabetic cardiomyopathy. *Circulation Research*.

[9] Wilson PWF (1998). Diabetes mellitus and coronary heart disease. *American Journal of Kidney Diseases*.

[10] Colhoun H (2006). Coronary heart disease in women: why the disproportionate risk?. *Current Diabetes Reports*.

[11] Kuncová J, Švíglerová J, Tonar Z, Slavíková J (2005). Heterogenous changes in neuropeptide Y, norepinephrine and epinephrine concentrations in the hearts of diabetic rats. *Autonomic Neuroscience*.

[12] Garofano A, Czernichow P, Breant B (2000). Impaired *β*-cell regeneration in perinatally malnourished rats: a study with STZ. *The Federation of American Societies for Experimental Biology Journal*.

[13] Su EN, Alder VA, Yu DY, Yu PK, Cringle SJ, Yogesan K (2000). Continued progression of retinopathy despite spontaneous recovery to normoglycemia in a long-term study of streptozotocin-induced diabetes in rats. *Graefe’s Archive for Clinical and Experimental Ophthalmology*.

[14] Rossini AA, Williams RM, Appel MC, Like AA (1978). Sex differences in the multiple-dose streptozotocin model of diabetes. *Endocrinology*.

[15] Leiter EH (1982). Multiple low-dose streptozotocin-induced hyperglycemia and insulitis in C57BL mice: influence of inbred background, sex, and thymus. *Proceedings of the National Academy of Sciences of the United States of America*.

[16] Hicks KK, Seifen E, Stimers JR, Kennedy RH (1998). Effects of streptozotocin-induced diabetes on heart rate, blood pressure and cardiac autonomic nervous control. *Journal of the Autonomic Nervous System*.

[17] Alings AMW, Bouman LN (1993). Electrophysiology of the ageing rabbit and cat sinoatrial node: a comparative study. *European Heart Journal*.

[18] Dobrzynski H, Boyett MR, Anderson RH (2007). New insights into pacemaker activity: promoting understanding of sick sinus syndrome. *Circulation*.

[19] Jones SA, Boyett MR, Lancaster MK (2007). Declining into failure: the age-dependent loss of the L-type calcium channel within the sinoatrial node. *Circulation*.

[20] Christou DD, Seals DR (2008). Decreased maximal heart rate with aging is related to reduced *β*-adrenergic responsiveness but is largely explained by a reduction in intrinsic heart rate. *Journal of Applied Physiology*.

[21] Howarth FC, Nowotny N, Zilahi E, El Haj MA, Lei M (2007). Altered expression of gap junction connexin proteins may partly underlie heart rhythm disturbances in the streptozotocin-induced diabetic rat heart. *Molecular and Cellular Biochemistry*.

[22] Howarth CF, Al-Sharhan R, Al-Hammadi A, Qureshi MA (2007). Effects of streptozotocin-induced diabetes on action potentials in the sinoatrial node compared with other regions of the rat heart. *Molecular and Cellular Biochemistry*.

[23] Linnemann B, Janka HU (2003). Prolonged QTc interval and elevated heart rate identify the type 2 diabetic patient at high risk for cardiovascular death. The Bremen diabetes study. *Experimental and Clinical Endocrinology and Diabetes*.

[24] Larsen JA, Kadish AH (1998). Effects of gender on cardiac arrhythmias. *Journal of Cardiovascular Electrophysiology*.

[25] Pham TV, Rosen MR (2002). Sex, hormones, and repolarization. *Cardiovascular Research*.

[26] Cheng J (2006). Evidences of the gender-related differences in cardiac repolarization and the underlying mechanisms in different animal species and human. *Fundamental and Clinical Pharmacology*.

[27] Felten SY, Peterson RG, Shea PA, Besch HR, Felten DL (1982). Effects of streptozotocin diabetes on the noradrenergic innervation of the rat heart: a longitudinal histofluorescence and neurochemical study. *Brain Research Bulletin*.

[28] Yoshida T, Nishioka H, Nakamura Y, Kondo M (1985). Reduced noradrenaline turnover in streptozotocin-induced diabetic rats. *Diabetologia*.

[29] Akiyama N, Okumura K, Watanabe Y (1989). Altered acethlcholine and norepinephrine concentrations in diabetic rat hearts. Role of parasympathetic nervous system in diabetic cardiomyopathy. *Diabetes*.

[30] Ganguly PK, Beamish RE, Dhalla KS, Innes IR, Dhalla NS (1987). Norepinephrine storage, distribution, and release in diabetic cardiomyopathy. *American Journal of Physiology*.

[31] Gando S, Hattori Y, Kanno M (1993). Altered cardiac adrenergic neurotransmission in streptozotocin-induced diabetic rats. *British Journal of Pharmacology*.

[32] Snyder DL, Aloyo VJ, Wang W, Roberts J (1998). Influence of age and dietary restriction on norepinephrine uptake into cardiac synaptosomes. *Journal of Cardiovascular Pharmacology*.

[33] Takenouchi Y, Kobayashi T, Taguchi K, Matsumoto T, Kamata K (2010). Gender differences in vascular reactivity of aortas from streptozotocin-induced diabetic mice. *Biological and Pharmaceutical Bulletin*.

[34] Kuncová J, Slavíková J, Švíglerová J (2003). Norepinephrine release in the heart atria of diabetic rats. *General Physiology and Biophysics*.

[35] Trendelenburg U (1990). Carrier-mediated outward transport of noradrenaline from adrenergic varicosities. *Polish Journal of Pharmacology and Pharmacy*.

[36] Kiyono Y, Kajiyama S, Fujiwara H, Kanegawa N, Saji H (2005). Influence of the polyol pathway on norepinephrine transporter reduction in diabetic cardiac sympathetic nerves: implications for heterogeneous accumulation of MIBG. *European Journal of Nuclear Medicine and Molecular Imaging*.

[37] Zugck C, Lossnitzer D, Backs J, Kristen A, Kinscherf R, Haass M (2003). Increased cardiac norepinephrine release in spontaneously hypertensive rats: role of presynaptic alpha-2A adrenoceptors. *Journal of Hypertension*.

[38] Richardt D, Dendorfer A, Tölg R, Dominiak P, Richardt G (2006). Inhibition of nonexocytotic norepinephrine release by desipramine reduces myocardial infarction size. *Canadian Journal of Physiology and Pharmacology*.

[39] Langeloh A, Bonisch H, Trendelenburg U (1987). The mechanism of the 3H-noradrenaline releasing effect of various substrates of uptake1: multifactorial induction of outward transport. *Naunyn-Schmiedeberg’s Archives of Pharmacology*.

[40] Levi G, Raiteri M (1993). Carrier-mediated release of neurotransmitters. *Trends in Neurosciences*.

[41] Javorková V, Mézešová L, Vlkovičová J, Vrbjar N (2010). Influence of sub-chronic diabetes mellitus on functional properties of renal Na(+),K(+)-ATPase in both genders of rats. *General Physiology and Biophysics*.

